# The Importance of Studying Health Behavior in Older Adults

**DOI:** 10.1093/geroni/igaa057.3020

**Published:** 2020-12-16

**Authors:** Jaime Hughes, Susan Hughes, Mina Raj, Janet Bettger

**Affiliations:** 1 Duke University School of Medicine, Durham, North Carolina, United States; 2 University of Illinois at Chicago, Chicago, Illinois, United States; 3 University of Michigan, Ann Arbor, Michigan, United States

## Abstract

Behavior change is an inherent aspect of routine geriatric care. However, most research and clinical programs emphasis how to initiate behavior change with less emphasis placed on skills and strategies to maintain behaviors over time, including after an intervention has concluded. This presentation will provide an introduction to the symposium, including a review of prior work and our rationale for studying the critical yet overlooked construct of maintenance in older adults. Several key considerations in our work include the impact of multiple chronic conditions, declines in cognitive and functional capacity over time, changes in environmental context and/or social support, and sustainability of community and population-level programs and services.

